# Ultrasound characteristics of abdominal vascular compression syndromes

**DOI:** 10.3389/fcvm.2023.1282597

**Published:** 2023-12-18

**Authors:** Yan Liu, Haining Zheng, Xiaoqing Wang, Zi Wang, Qiang Zhu, Chaoyang Wen, Yisha Tong

**Affiliations:** ^1^ Department of Ultrasound, Peking University International Hospital, Beijing, China; ^2^ Department of Ultrasound, Beijing Tongren Hospital, Capital Medical University, Beijing, China; ^3^ Department of Vascular Surgery, Austin Hospital, University of Melbourne, Melbourne, VIC, Australia

**Keywords:** abdominal vascular compression syndrome, celiac artery compression syndrome, iliac vein compression syndrome, renal vein compression syndrome, superior mesenteric artery syndrome, ultrasound

## Abstract

Abdominal vascular compression syndrome (AVCS) is caused by the compression of abdominal blood vessels by adjacent structures or the compression of abdominal organs by neighboring blood vessels. Such compressions can result in a variety of clinical symptoms. They are not commonly seen in ultrasound practices, and their presence may have been underrecognized and underdiagnosed. This article reviews the clinical features, ultrasound characteristics, and diagnostic criteria of four types of AVCS, namely, celiac artery compression syndrome, renal vein compression syndrome, iliac vein compression syndrome, and superior mesenteric artery syndrome to increase awareness of these conditions among ultrasound practitioners. The ultrasound criteria for AVCS are primarily based on studies with small sample sizes, and therefore, it is important to exercise caution if these criteria are used.

## Introduction

Blood vessels in the abdomen may be compressed by adjacent anatomical structures, or they may cause compression of organs in the abdomen. Abdominal vascular compression phenomena are referred to the identification of such compressions with or without clinical symptoms. When the compression causes relevant clinical symptoms, it is regarded as an abdominal vascular compression syndrome (AVCS) (1–5).

AVCS involving the compression of blood vessels includes celiac artery compression syndrome (CACS), renal vein compression syndrome (RVCS), and iliac vein compression syndrome (IVCS). AVCS involving the compression of organs consists of superior mesenteric artery syndrome (SMAS), ureteropelvic junction obstruction, and retrocaval ureter (Table 1) (3, 6, 7).

**Table 1:** Abdominal vascular compression syndromes.

Syndrome	Also known as	Compressed structure	Cause of compression	Clinical manifestations
Celiac artery compression syndrome	Median arcuate ligament syndrome Dunbar syndrome	Celiac artery	Median arcuate ligament	Epigastric pain, weight loss, nausea, and vomiting
Renal vein compression syndrome	left renal vein entrapment syndrome Nutcracker syndrome	Left renal vein	SMA and AA (anterior nutcracker) AA and vertebral body (posterior nutcracker)	Left flank pain, hematuria, symptoms associated with gonadal vein reflux
Iliac vein compression syndrome	May–Thurner syndrome Cockett syndrome	Left CIV	Right CIA and vertebral body	Left lower limb swelling, symptoms associated with deep vein thrombosis
Superior mesenteric artery syndrome	Cast syndrome Wilkie syndrome	Transverse duodenum	SMA and AA	Postprandial epigastric pain, weight loss, nausea, and vomiting
Ureteropelvic junction obstruction		Ureteropelvic junction	Crossing renal artery or vein	Flank pain, pyelonephritis, hematuria
Retrocaval ureter		Proximal ureter	IVC and vertebral body	Flank pain, hematuria

SMA, superior mesenteric artery; AA, abdominal aorta; CIV, common iliac vein; CIA, common iliac artery.

AVCS are uncommon, and perhaps underrecognized and underdiagnosed. We review the clinical features, ultrasound characteristics, and diagnostic criteria of the first four types of AVCS to help sonographers and sonologists become familiar with these conditions and encourage them to take them into consideration when making diagnostic assessments in their ultrasound practice. Of note, all of these syndromes are primarily observed in young and middle-aged women (3, 4, 8–10).

The last two AVCS are not included in this review due to the limited information on their ultrasound findings in the literature.

## Celiac artery compression syndrome

**Figure 1:**
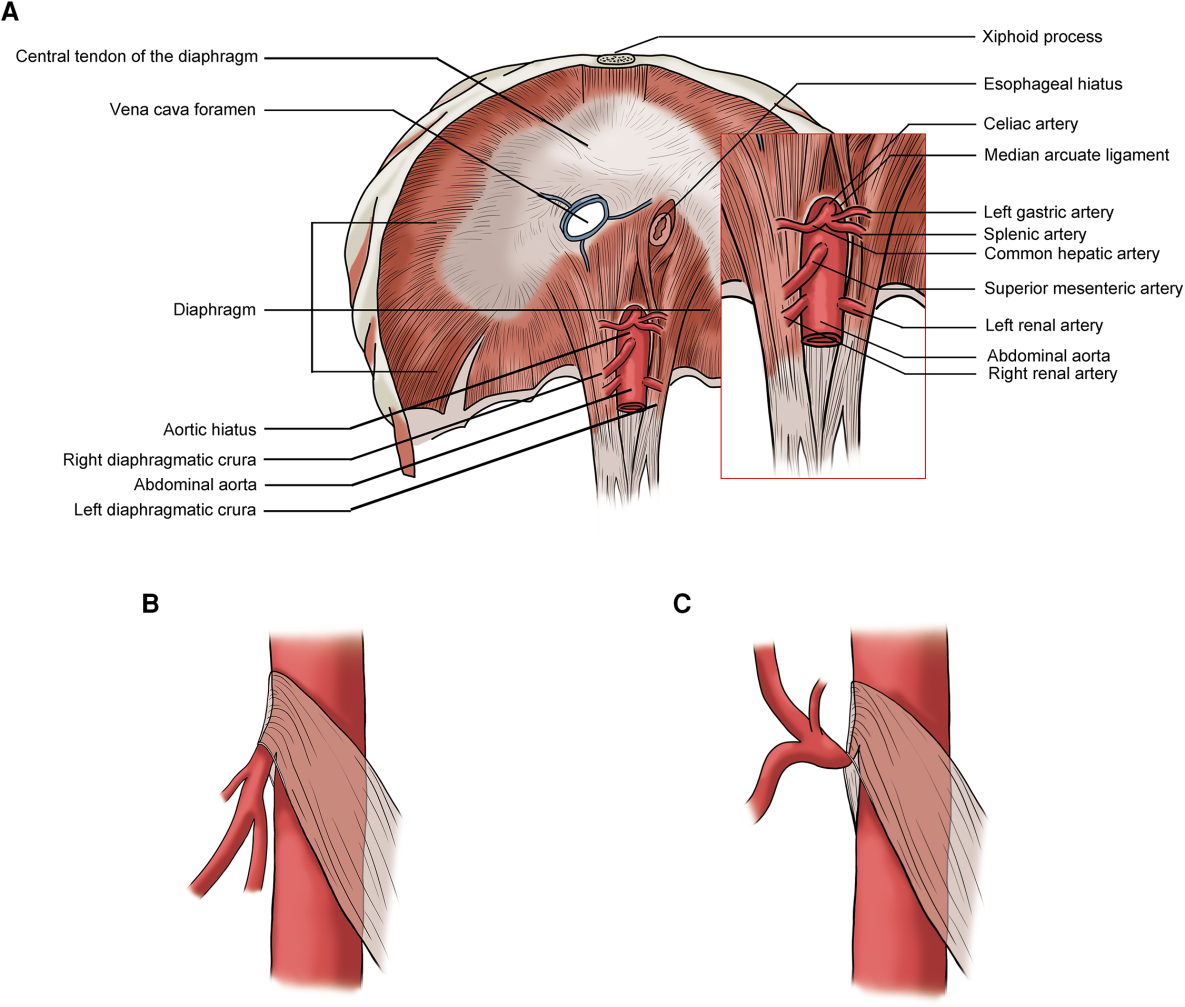
(**A**) Normal median arcuate ligament (MAL) position; (**B**) lower MAL position: no significant celiac artery compression during inspiration; (**C**) lower MAL position: celiac artery compressed by the MAL during expiration.

The celiac artery compression syndrome (CACS), also known as median arcuate ligament syndrome (MALS) and Dunbar syndrome, is caused by the external compression of the median arcuate ligament (MAL) on the celiac artery (CA). The MAL is the fibrous arc of the aortic hiatus in the diaphragm which connects the left and right diaphragmatic crura and usually lies superior to the origin of celiac artery (Figure 1A). When the MAL has a low insertion, it can compress the celiac artery. The compression usually worsens during expiration as the diaphragm moves caudally (Figures 1B,C) (8, 11). Patents with MAL compression may have no symptoms. Postprandial abdominal pain and weight loss are the most common symptoms in symptomatic patients with CACS. Other symptoms include nausea and vomiting (3). Most CACS are diagnosed when other chronic abdominal pain-related diseases are excluded.

Radiological examinations including computed tomography angiography (CTA), magnetic resonance angiography (MRA), and selective angiography can be used to confirm the presence of MAL compression (7, 12, 13). It is important to assess the CA with inspiration along with expiration as CA compression may only occur at the end of the expiration. CT and MRI may also be used to rule out other causes of CA compression (14) such as lymph node metastases.

Many patients with CACS are treated using conservative management approaches. The surgical options for CACS include open or laparoscopic surgery to relieve MAL compression, open or endovascular revascularization of the CA, and celiac ganglion resection (3, 10, 11). More recently, robotic-assisted MAL release has been used in patients with MALS (15).

### Ultrasound characteristics and diagnostic criteria

**Figure 2:**
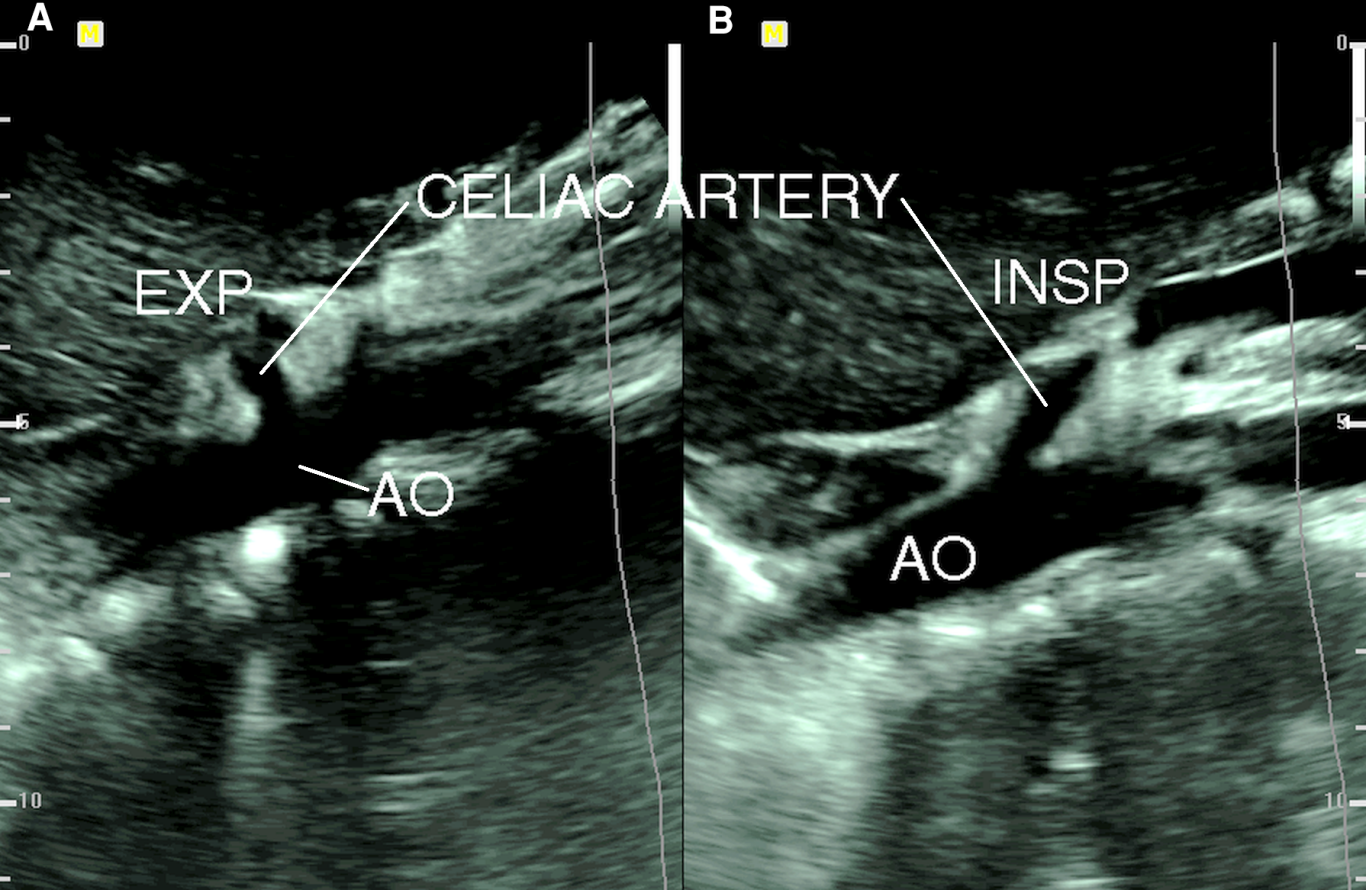
B-mode ultrasound image of celiac artery compression. (**A**) Celiac artery deflection angle > 50° during expiration (EXP); (**B**) celiac artery deflection angle < 50° during inspiration (INSP). AO, aorta [from: Youssef (18). Reprint with permission from Dr Youssef].

Gruber and colleagues (16) compared the findings of duplex ultrasound in six patients with CACS with 20 age-matched asymptomatic volunteers and found that a peak systolic velocity (PSV) of the CA during expiration greater than 350 cm/s had an 83% positive predictive value (PPV) and a 95% negative predictive value (NPV). The CA deflection angle (end-expiratory upturn angle) of 50° or more has a PPV of 43% and an NPV of 100%, and the combination of CA PSV (>350 cm/s) and deflection angle (>50°) has a PPV of 100% and NPV of 95%. Saleem and coauthors (17) described the ultrasound criteria that are supportive in diagnosing CACS, which include expiratory CA PSV of > 200 cm/s and a CA deflection angle of > 50°. The changes of CA deflection angle during inspiration and expiration in a patient with CACS are shown in Figure 2. Another ultrasound criterion for diagnosing CACS includes measuring the PSV of the CA during the expiratory phase. A PSV of over 200 cm/s or a ratio of PSV of CA to aorta of greater than 3:1 in the expiratory phase can also indicate CACS (18–23). In patients with an optimal sonographic window, the “hook” or “J” shape of the proximal CA caused by extrinsic compression by the MAL can be demonstrated in the sagittal plane with a B-mode or color Doppler imaging (24).

The use of intravascular ultrasound (IVSU) to assess patients with MALS has been reported to show substantial luminal stenosis at the origin of the celiac artery with wall thickening (25). This finding is suggestive of CA intimal hyperplasia from chronic irritation by the MAL.

## Renal vein compression syndrome

**Figure 3:**
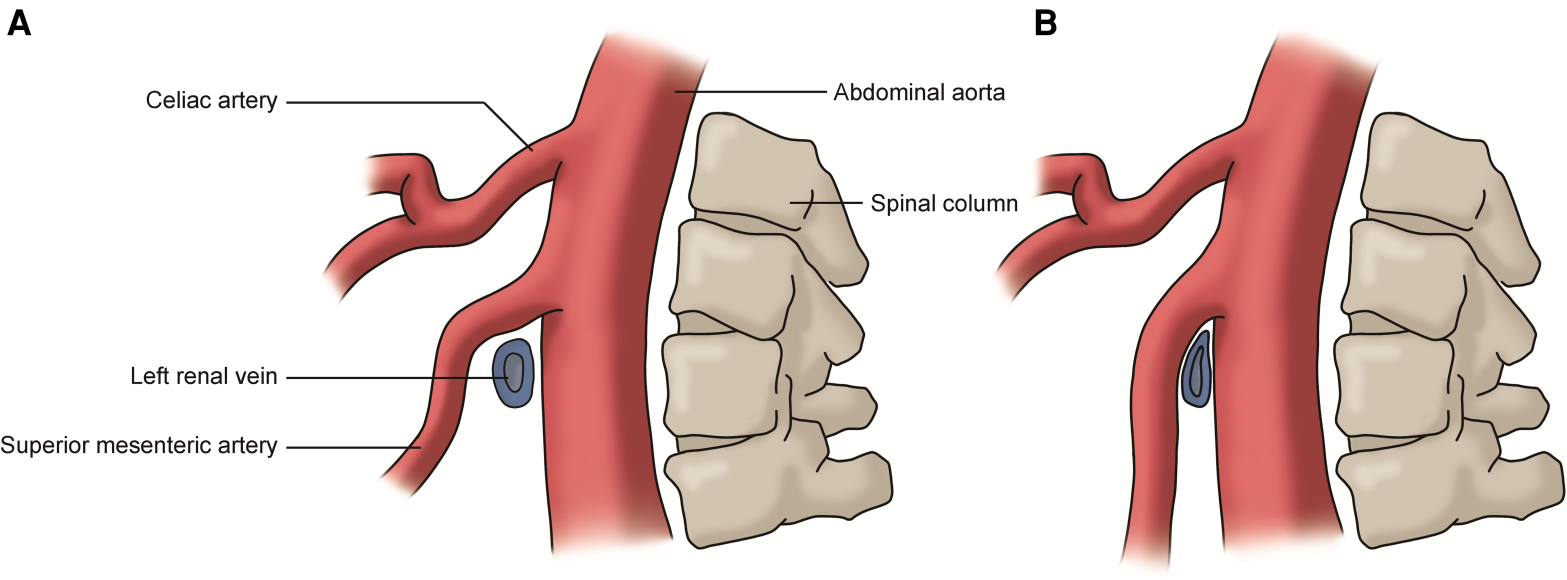
(**A**) Normal left renal vein: the vein passes between the superior mesenteric artery and abdominal aorta without compression; (**B**) renal vein compression syndrome: the left renal vein is compressed by the superior mesenteric artery and the abdominal aorta.

The renal vein compression syndrome (RVCS), also known as left renal vein entrapment syndrome and nutcracker syndrome (NCS), refers to the compression of the left renal vein (LRV) by the superior mesenteric artery (SMA) and abdominal aorta (AA), like the compression of a nut between the jaws of a nutcracker (Figure 3) (26). This type of RVCS is also known as the anterior nutcracker syndrome (ANCS). In addition, when the retroaortic LRV is compressed by the AA and the vertebral body, it is referred to as the posterior nutcracker syndrome (PNCS) (27). There is also a known anatomical variant involving two LRVs that pass anteriorly and posteriorly to the AA. In these patients, the simultaneous compression of both veins is referred to as the combined nutcracker syndrome (14).

The clinical manifestations of the RVCS include left flank pain, hematuria, proteinuria, and symptoms related to left gonadal vein reflux such as pelvic congestion syndrome in the female and varicocele in the male. CT venography and contrast venography are useful radiological modalities for diagnosis (3, 26, 28). The conservative management involves gaining weight and exercising the back muscles. Open surgical options such as renocaval bypass and transposition of the LRV to the inferior vena cava and endovascular interventions including stent implantation and ovarian vein embolization are used when conservative management has failed (3, 29). Laparoscopic extravascular stent placement has been reported as an alternative to open surgery or endovascular intervention (30).

### Ultrasound characteristics and diagnostic criteria

**Figure 4:**
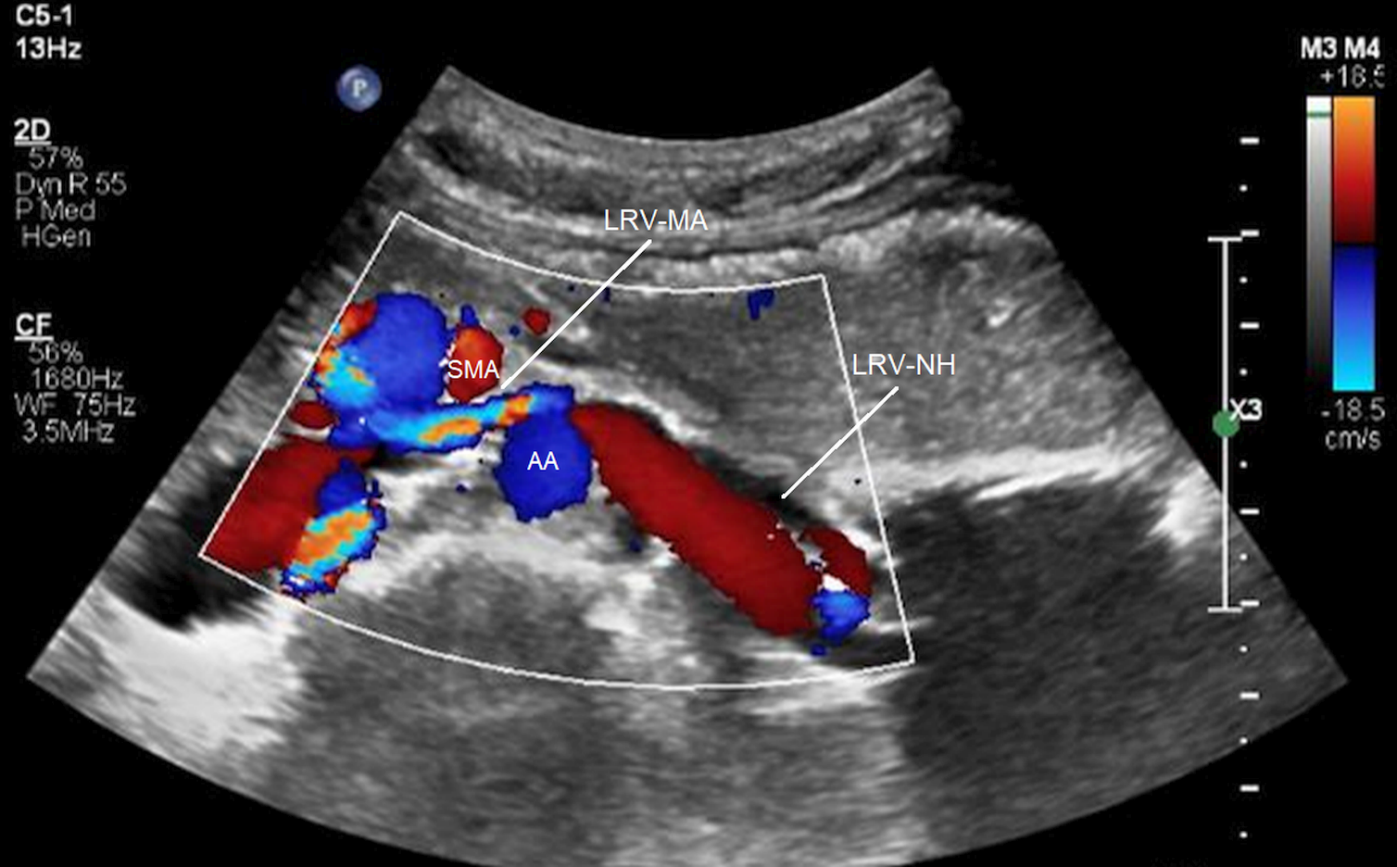
Compression of the left renal vein on color Doppler imaging. SMA, superior mesenteric artery; AA, abdominal aorta; LRV-MA, left renal vein between the SMA and AA; LRV-NH, left renal vein near the hilar [from: Kim (32). Reprinted with permission from Dr Kim].

Kim and coworkers (31, 32) analyzed duplex ultrasound results of the left renal vein in 16 patients with RVCS and 18 healthy control subjects. They measured the diameters and PSVs in two regions of the left renal vein: firstly, between the SMA and the aorta (MA) and secondly, near the hilar (NH) (Figure 4). They recommended ultrasound criteria for RVCS of diameter ratio (NH/MA) > 5 (sensitivity: 69% and specificity: 89%) and PSV ratio (MA/NH) > 5 (sensitivity: 80% and specificity: 94%). Yang and colleagues (33) reported eight cases of PNCS and described their ultrasound diagnostic criteria for PNCS as follows: firstly, the finding of a retroaortic left renal vein; secondly, the diameter of the dilated part of the left renal vein (D-VD): diameter of stenotic part of the vein (S-VD) >3:1 in the supine position, or >4:1 in the spinal extension position; and thirdly, the PSV in the stenotic part of the left renal vein: PSV in the dilated part of the vein >3:1, or >4:1 in the spinal extension position.

**Figure 5:**
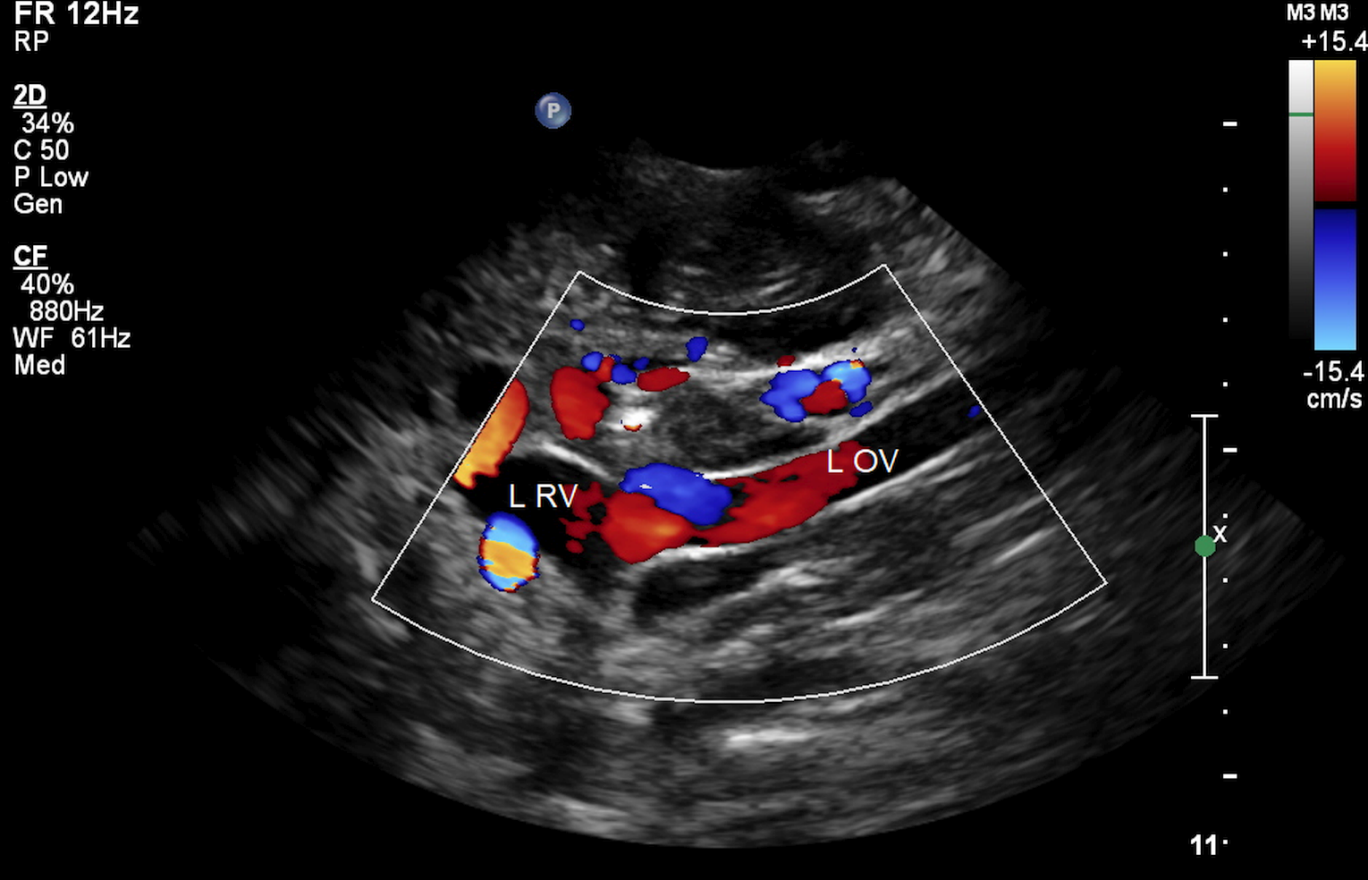
Ovarian vein reflux on color Doppler imaging. L RV, left renal vein; L OV, left ovarian vein.

The left gonadal vein is a tributary of the LRV. LRV hypertension may lead to incompetence of the left gonadal vein (34). Left ovarian vein reflux in a patient with RVCS is shown in Figure 5.

## Iliac vein compression syndrome

**Figure 6:**
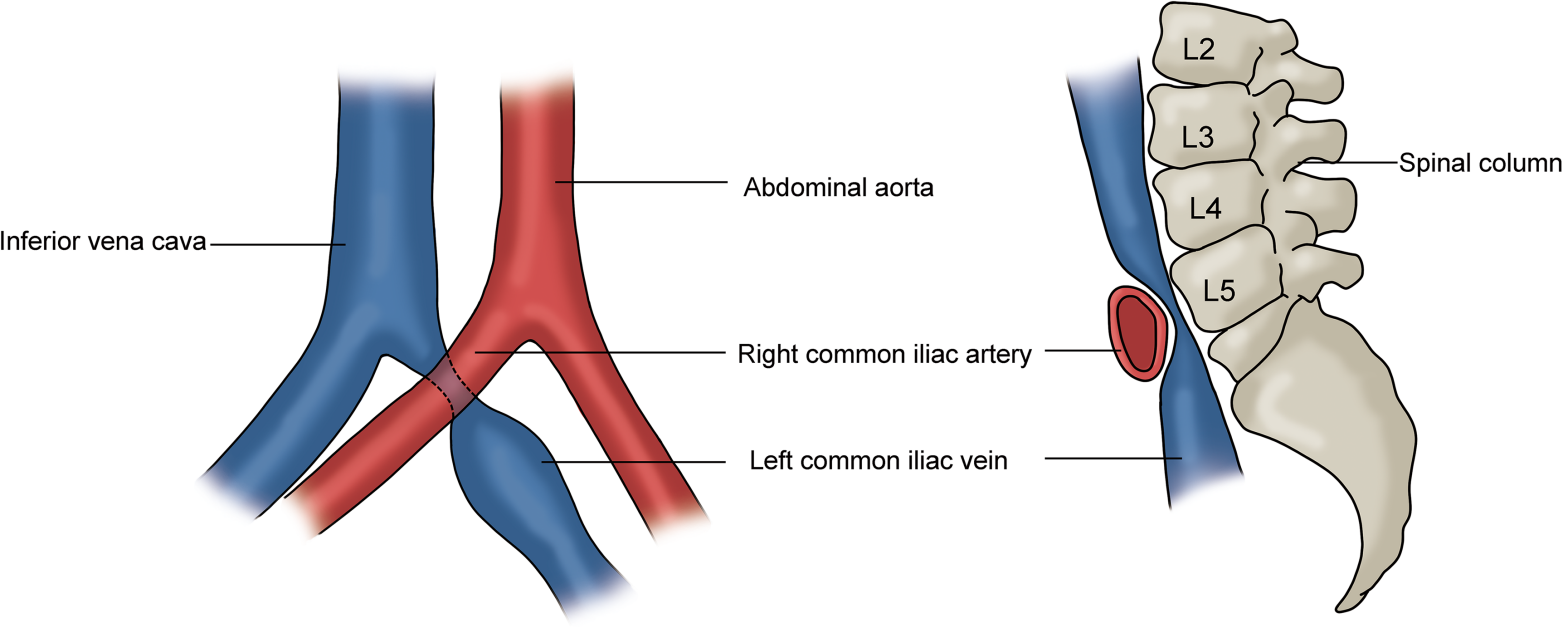
Iliac vein compression syndrome. Left: the left common iliac vein is compressed by the right common iliac artery; Right: the compressed left CIV is located between the right common iliac artery and the vertebral body.

The iliac vein compression syndrome (IVCS), also known as May–Thurner syndrome (MTS) and Cockett syndrome, is a clinical condition caused by the extrinsic compression of the left common iliac vein (CIV) by the right common iliac artery (CIA) and vertebral body (Figure 6) (9). The clinical symptoms include swelling of the left lower limb, edema, and varicose veins. In the event of the development of deep vein thrombosis (DVT), symptoms related to acute DVT, post-thrombotic syndrome, and pulmonary embolism may result (3, 9, 35). CT/MR venography and contrast venography are diagnostic tools used to assess IVCS. However, contrast venography is mainly used in conjunction with intervention (3, 9, 36, 37). The conservative management includes the use of compression stockings and anticoagulation therapy. Iliac vein stenting and DVT thrombolysis may be considered when conservative treatment has failed (3).

### Ultrasound characteristics and diagnostic criteria

**Figure 7:**
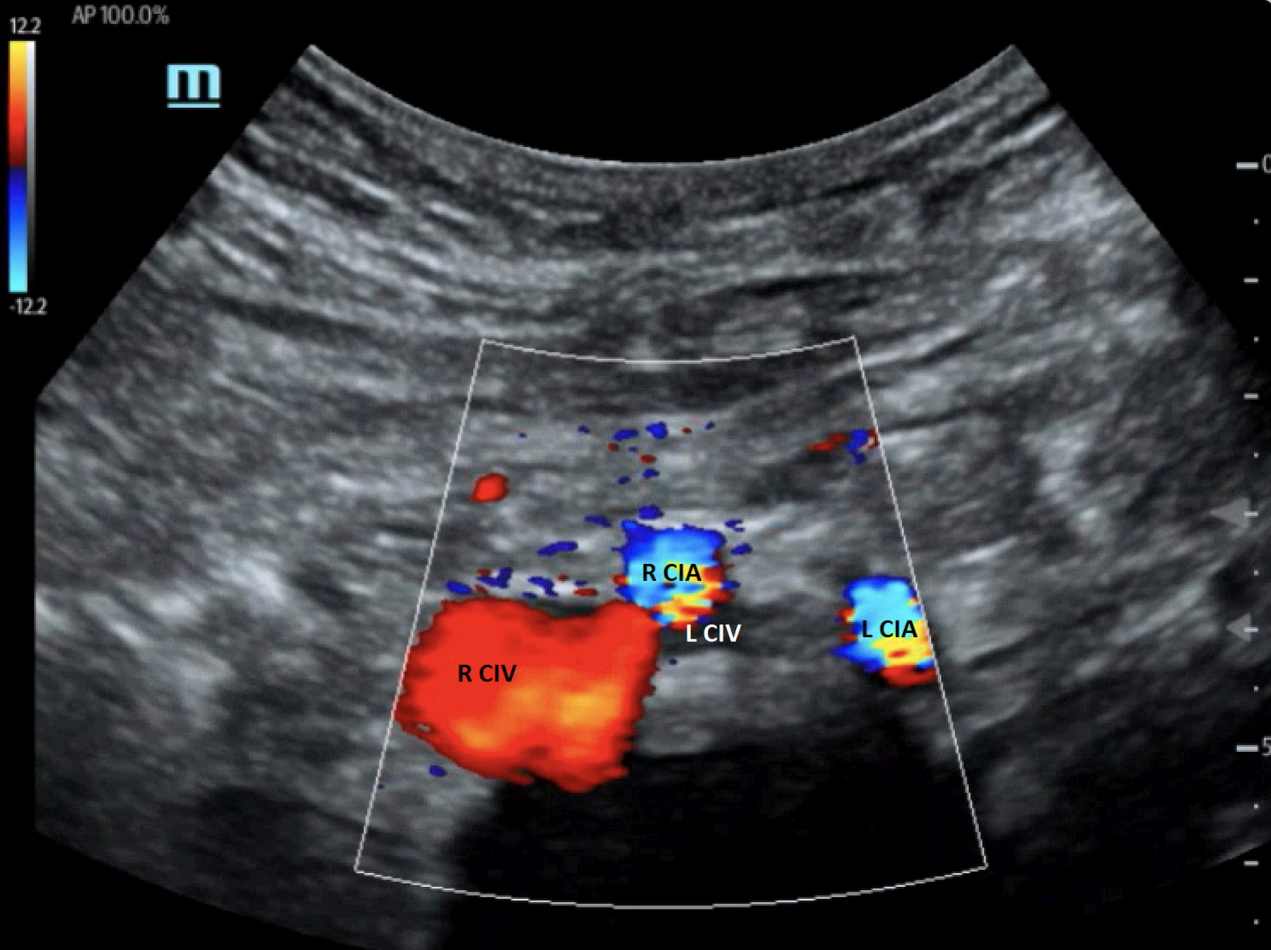
Color Doppler image of iliac vein compression syndrome. The left common iliac vein (L CIV) is compressed by the right common iliac artery (R CIA). R CIV, right common iliac vein; L CIA, left common iliac artery [From: Brown et al. (39). Reprint under a Creative Commons Attribution 4.0 International License].

The compression of the left CIV by the right CIA can be demonstrated with duplex ultrasound when a good acoustic window is obtained (38, 39). Figure 7 shows the anatomical relationship of the right and left CIAs and CIVs. In this image, the left CIV is compressed by the right CIA. However, direct visualization of a severe stenosis of the left CIV at the point that the right CIA crosses the vein is not always possible on B-mode ultrasound imaging. Duplex ultrasound may demonstrate venous flow without respiratory variation in the iliac vein below the compression point, and flow velocity increases at the location of the right CIA compressing the left CIV (40). If the PSV gradient is more than 2.5, the findings are significant. Flow reversal in the ipsilateral internal iliac vein can be associated with CIV compression (3, 41). The first clinical presentation of some patients with IVCS can be left iliac vein thrombosis. Confirming the presence of deep vein thrombosis may not be difficult, but demonstrating the left iliac vein compression is not generally possible when the vessel is thrombosed.

IVUS has been used successfully to detect iliac vein compression. It provides information about the spatial relationships of anatomic structures and compression of the left CIV by the right CIA in the case of IVCS. As the modality assesses the vessel wall directly, venous spurs thought to be caused by a combination of mechanical compression and arterial pulsations on the trapped vein can also be visualized (41–44).

## Superior mesenteric artery syndrome

**Figure 8:**
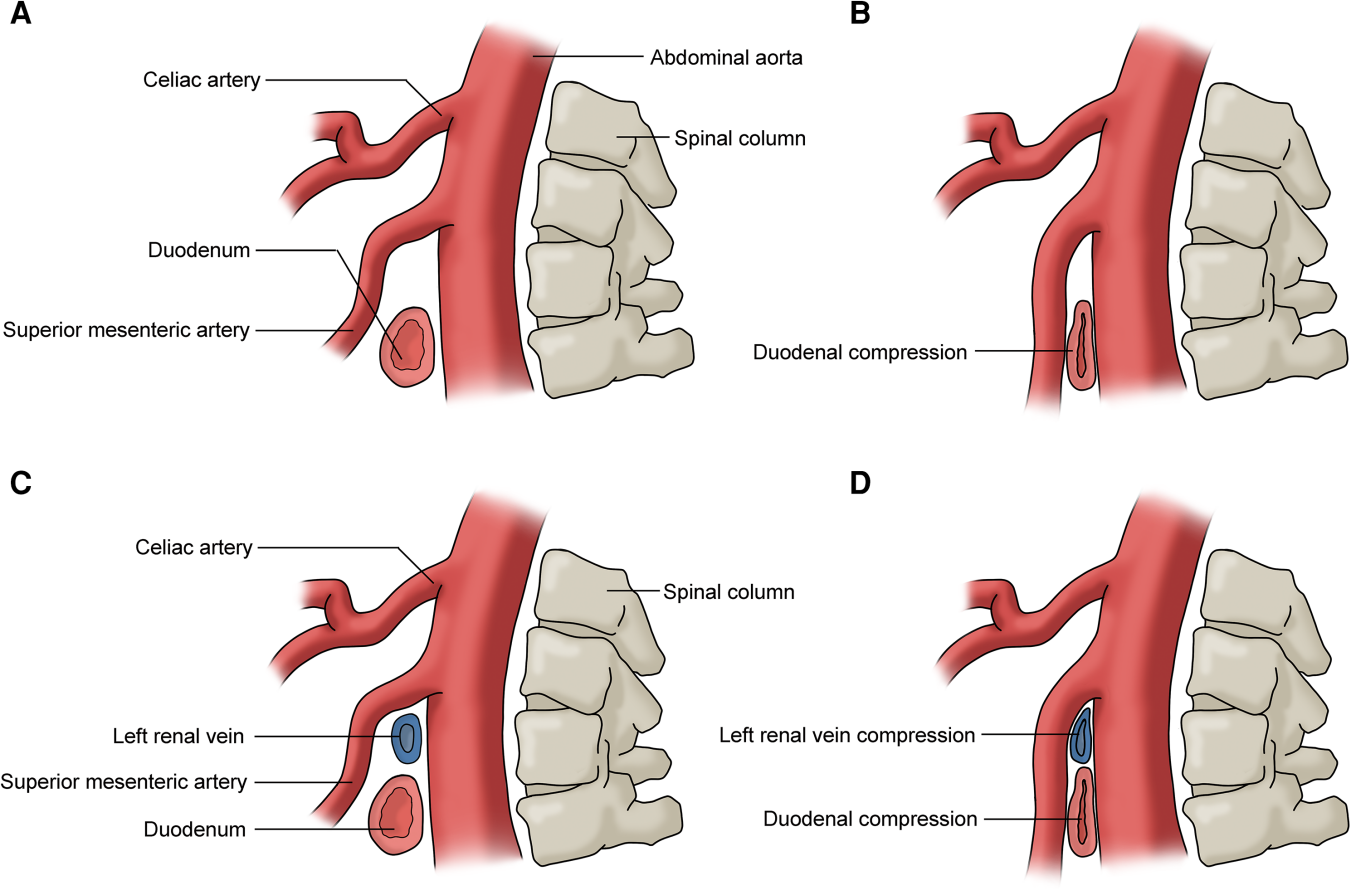
(**A**) No duodenum compression; (**B**) duodenum compression (superior mesmeric artery compression syndrome); (**C**) no duodenum or left renal vein compression; (**D**) duodenum and left renal vein compression (superior mesmeric artery compression syndrome and renal vein compression syndrome).

The superior mesenteric artery syndrome (SMAS), also known as Cast syndrome and Wilkie syndrome, is a vascular compression syndrome that presents clinically with upper small bowel obstruction due to entrapment of the third part of the duodenum between the SMA and AA (Figures 8A,B) (42). The common symptoms include postprandial epigastric pain, weight loss, nausea, and vomiting (3, 45). SMAS may be accompanied by RVCS as they share the same pathogenesis (Figures 8C,D) (46).

Barium testing, abdominal x-ray, CT/CTA, MRI/MRA, and endoscopy play important roles in the diagnosis and differential diagnosis of SMAS (47–49). The main therapeutic goal is eliminating obstructions and obtaining optimal weight gains. The initial management is usually conservative, including decompression of the duodenum with a nasogastric tube and nutritional support. Surgical interventions consist of Strong's procedure to release the Treitz ligament, gastrojejunostomy, or duodenojejunostomy to bypass the duodenum obstruction (3, 10).

### Ultrasound characteristics and diagnostic criteria

**Figure 9:**
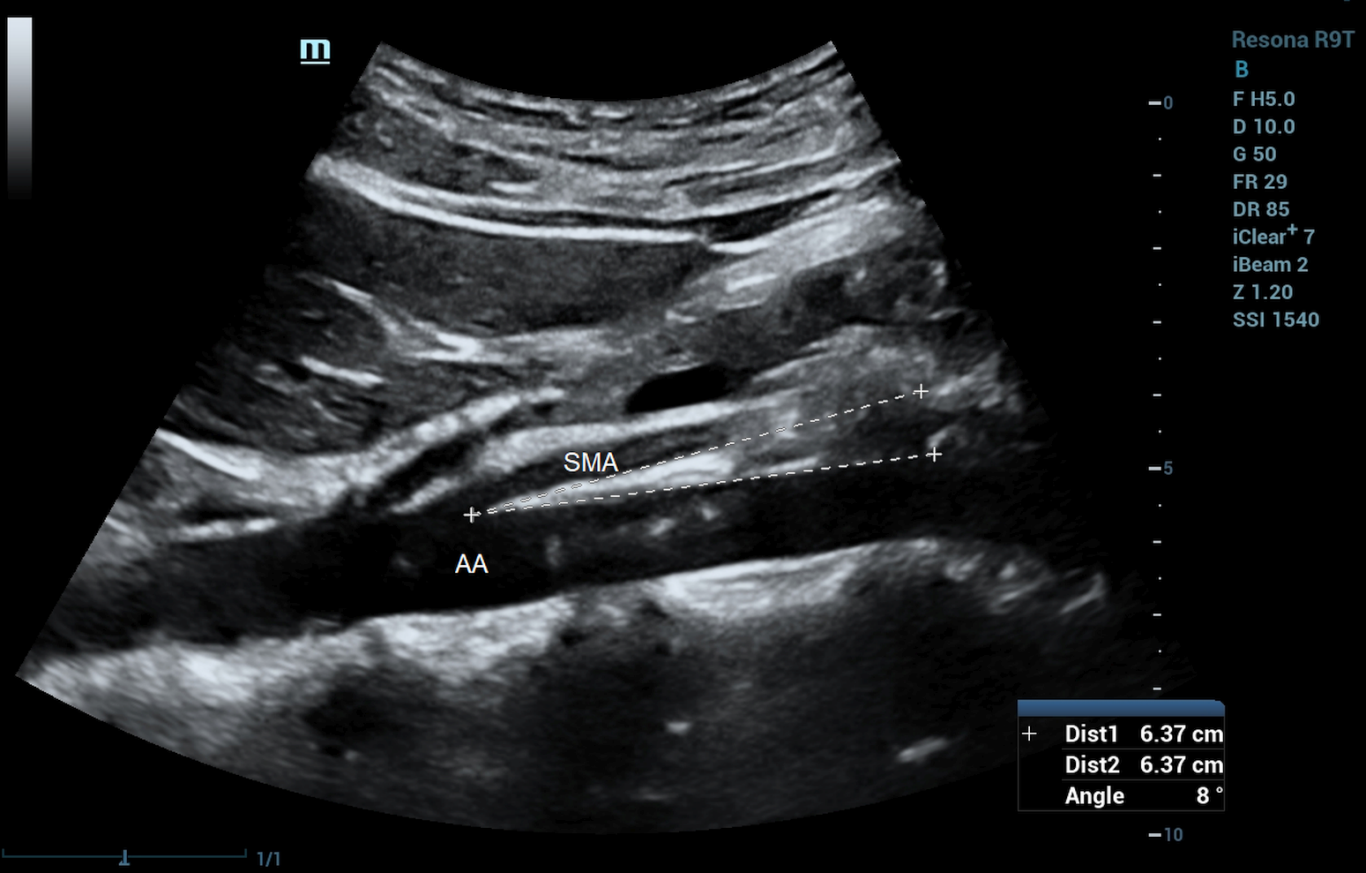
Superior mesenteric artery syndrome on B-mode imaging. Aorto-mesenteric angle = 8°. SMA, superior mesenteric artery; AA, abdominal aorta.

The ultrasound characteristics of SMAS are reduced SMA-AA distance and reduced SMA-AA angle. Unal and coworkers (50) reported cutoff values of 8 mm for SMA-AA distance (100% sensitivity and specificity), and 22° for SMA-AA angle (42.8% sensitivity, 100% specificity). Similar thresholds of aorto-mesenteric distance (<8 mm) and aorto-mesenteric angle (<25°) were reported by Neri and colleagues (51). Figure 9 shows a case of SMAS with an aorto-mesenteric angle of 8°. Mauceri et al. (52) demonstrated a statistically significant correlation between the use of power Doppler ultrasound and CT for detecting reduced aorto-mesenteric angle and suggested that duplex ultrasound can be used for screening of reduced aorto-mesenteric angle to diagnose suspected cases of SMAS in patients with inexplicable abdominal pain. The application of duplex ultrasound to assess for SMA syndrome has been carried out not only in the outpatient setting but also in the emergency department (53).

## Summary

The duplex ultrasound scan is a non-invasive, dynamic, and low-cost diagnostic examination. Ultrasound scanning is easily reproducible with the patient positioned in various postures or assessed during different respiratory phases. It provides morphological information regarding the anatomical structures and hemodynamic changes in the examined arteries or veins (3).

The duplex ultrasound plays an important role in the diagnosis of various vascular disorders and can be successfully used to assess AVCS. Many authors recommended ultrasound as a first line imaging modality to assist the diagnosis of AVCS (14, 16, 54, 55). The article provides a concise summary of the ultrasound characteristics and diagnostic criteria for AVCS, which may be useful to sonographers and sonologists who may not be familiar with these conditions. However, it is noted that most of the ultrasound criteria for AVCS are based on studies with relatively small sample sizes. Therefore, caution should be taken when these criteria are used.
